# Substance-P Inhibits Cardiac Microvascular Endothelial Dysfunction Caused by High Glucose-Induced Oxidative Stress

**DOI:** 10.3390/antiox10071084

**Published:** 2021-07-05

**Authors:** Do Young Kim, Jiyuan Piao, Hyun Sook Hong

**Affiliations:** 1 Department of Biomedical Science and Technology, Graduate School, Kyung Hee University, Seoul 02447, Korea; a0az515@khu.ac.kr; 2 Department of Genetic Engineering, College of Life Science and Graduate School of Biotechnology, Kyung Hee University, Yong In 17104, Korea; piaojiyuan@khu.ac.kr; 3 East-West Medical Research Institute, Kyung Hee University, Seoul 02447, Korea

**Keywords:** hyperglycemia, oxidative stress, substance-P, cardiac microvascular endothelial cells

## Abstract

Diabetes is characterized by high glucose (HG) levels in the blood circulation, leading to exposure of the vascular endothelium to HG conditions. Hyperglycemia causes oxidative stress via excessive reactive oxygen species (ROS) production in the endothelium, which leads to cellular dysfunction and the development of diabetic vascular diseases. Substance-P (SP) is an endogenous peptide involved in cell proliferation and migration by activating survival-related signaling pathways. In this study, we evaluated the role of SP in cardiac microvascular endothelial cells (CMECs) in HG-induced oxidative stress. CMECs were treated with diverse concentrations of glucose, and then the optimal dose was determined. Treatment of CMECs with HG reduced their viability and induced excessive ROS secretion, inactivation of PI3/Akt signaling, and loss of vasculature-forming ability in vitro. Notably, HG treatment altered the cytokine profile of CMECs. However, SP treatment inhibited the HG-mediated aggravation of CMECs by restoring viability, free radical balance, and paracrine potential. SP-treated CMECs retained the capacity to form compact and long stretching-tube structures. Collectively, our data provide evidence that SP treatment can block endothelial dysfunction in hyperglycemia and suggest the possibility of using SP for treating diabetic complications as an antioxidant.

## 1. Introduction

Vascular complications are the leading cause of morbidity and mortality in patients with severe disease [1,2,3]. Functional impairment of the vascular endothelium has been observed in all forms of cardiovascular disease and in individuals with insulin resistance, obesity, and type 2 diabetes. Diabetes is linked to a number of vascular complications, including retinopathy, nephropathy, atherosclerosis, and coronary heart disease. These complications may mainly develop due to the presence of excessive glucose in the blood, called hyperglycemia.

Chronic hyperglycemia is a characteristic of diabetes and is the main cause of multiple complications associated with the disease. Although many aspects of the pathophysiology of diabetes are still unclear, it has been established that hyperglycemia contributes to the development of vascular dysfunction in diabetes [4]. The maintenance of hyperglycemia directly induces vascular injury in patients with diabetes [5,6,7,8]. In hyperglycemia conditions, vascular endothelial cells generate large amounts of reactive oxygen species (ROS) and lack reactive nitrogen species (RNS), such as nitric oxide (NO). This condition creates an imbalance between vasodilation and vasoconstriction, and creates oxidative stress, leading to the development of endothelial dysfunction. Dysfunctional endothelial cells are characterized by a low viability, poor migration capacity, and abnormal extracellular matrix, which eventually accelerate the progression of pathological diseases. Thus, HG-mediated oxidative stress contributes to the development of diabetic vascular disorders, and the inhibition of oxidative stress-induced injury is considered a therapeutic strategy for diabetes and its complications.

The principal role of protein kinase B (Akt) involves facilitating growth factor-mediated cell survival and blocking apoptotic cell death [9]. Akt is regulated by oxidative stress for cell survival [10] and phosphorylates IκB kinase (IKK) α/β. Activated IKKα/β mediates the nuclear translocation of nuclear factor kappa-light-chain-enhancer of activated B cell-dependent pro-survival genes [11]. Sustained oxidative stress affects the maintenance of active Akt signaling, thereby causing cell death. The ability to maintain an optimal level of active Akt is important for cellular protection under diverse stress conditions.

Cardiac microendothelial cells (CMECs) play an important role in the physiological regulation of coronary blood flow and capillary exchange under normal physiological conditions. Under acute stress, endothelial cells create a physical environment that is favorable for remission of the injury in the body. However, sustained stimulation of excessive stress irreversibly induces endothelial dysfunction. Chronic hyperglycemia and inflammation are representative stresses that induce CMEC malfunction and apoptosis [12]. Microvascular endothelial dysfunction has been clearly observed in heart failure associated with type 2 diabetes and has been proposed to mediate myocardial dysfunction [13,14]. In this case, endothelial cells show reduced NO synthesis, increased production of ROS, and inflammatory features [15]. Cardiomyopathy has also been observed to be mainly accompanied by CMEC injury and cardiomyocyte dysfunction [16]. Therefore, cardiac microvascular endothelial impairment is closely related to the homeostasis of cardiac function, and the protection of CMECs against oxidative stress is expected to inhibit the pathogenesis of diabetic cardiovascular disease.

In a clinical trial, the control of blood glucose in patients with diabetes was attempted via treatment with anti-hyperglycemic drugs. The serious aspects of diabetes are its complications; patients with diabetes showed macro/microvascular complications [17,18]. However, conventional therapy for diabetes is unable to directly reduce oxidative stress, because it targets glucose uptake and has no antioxidant activity. Antioxidant therapy is estimated to be effective in diabetic conditions, possibly by protecting tissues against oxidative stress. Previous studies corroborated that antioxidant therapy successfully decreases ROS levels and restores endothelial dysfunction in animal models and in patients with diabetes [19,20,21]. However, the therapeutic efficiency of the commonly used antioxidants is lower than that required, as seen in clinical trials. This suggests the necessity for the development of new antioxidant approaches to block vascular injury from oxidative stress.

Substance-P (SP) is an endogenous neuropeptide expressed in diverse cell types, including immune cells and neuronal cells. SP exerts its effects by binding neurokinin receptor 1 to signaling molecules via the G-protein coupled receptor pathway [22,23]. SP mediates the mobilization of stem cells into injured sites by repopulating stem cells residing in the bone marrow [24]. This function can accelerate tissue repair by promoting the participation of stem cells in wound healing. Moreover, SP can protect diverse types of cells against inflammation or oxidative stress by preserving cell viability and blocking cellular alterations [25,26,27]. The treatment of acute inflammatory disease with SP ameliorates excessive inflammation by increasing the number of M2 macrophages and regulatory T cells in the circulation and lymphoid organs, which reduces disease severity [28,29,30,31,32,33]. Additionally, SP can restore the cellular function of senescent stem cells and preserve paracrine potentials [4].

Based on the functioning of SP investigated in previous studies, we hypothesized that SP may be capable of recovering endothelial cells injured by HG-mediated oxidative stress. To determine the role of SP in the treatment of damaged endothelial cells, human CMECs were exposed to HG and SP. The effects of SP on impaired CMECs were assessed by evaluating cell viability, ROS/RNS production, paracrine factors, tube structure-forming capacity, and early signaling molecules.

## 2. Materials and Methods

### 2.1. Materials

SP and Phenylmethylsulfonyl fluoride (PMSF) were purchased from Sigma-Aldrich (St. Louis, MO, USA). Endothelial cell growth basal medium-2 MV was purchased from Lonza (Basel, Switzerland). Penicillin/streptomycin (P/S), 0.25% trypsin-ethylenediaminetetraacetic acid solution, and phosphate-buffered saline (PBS) were provided by Welgene (Daegu, Korea). WST-1 was purchased from Roche (Indianapolis, IN, USA). Cell lysis buffer, anti-Akt, anti-phospho-Akt, anti-eNOS, anti-phospho-eNOSser1177, anti-GSK-3β, and anti-phospho-GSK-3β antibodies were purchased from Cell Signaling Technology (Danvers, MA, USA). Anti-GAPDH antibody and cellular ROS assay kit were purchased from Abcam (Cambridge, MA, USA). Griess Reagent System was purchased from Promega (Madison, WI, USA). Matrigel was purchased from Corning (Corning, NY, USA). μ-slide was purchased from Ibidi Technology (Grafelfing, Germany). Human angiogenesis cytokine array kit was purchased from R&D Systems (Minneapolis, MN, USA).

### 2.2. Cell Culture

The human cardiac microvascular endothelial cells (CMECs) were obtained from (Lonza). The human cardiac microvascular endothelial cells were cultured in EGM-2 MV (Lonza) at 37 °C with 5% CO_2_. The medium was changed once every two days. The images were obtained using microscopy (Nikon Eclipse, Tokyo, Japan).

### 2.3. Glucose Exposure and SP Treatment

For high glucose exposure of the CMECs, CMECs were cultured in 6.25, 12.5, and 25 mM glucose. CMEC culture media (EGM-2 MV) was used as the control condition (glucose concentration: 2.7 mM). SP was added to CMEC in a final dose of 100 nM. PBS was treated as the control for SP treatment.

### 2.4. Wst-1 Assay

The CEMC (1.0 × 10^4^ cells/well) were seeded onto 96 well plates. For determining the viability, 20 uL of WST-1 solution were added to each well and the plate was incubated for 60 min at 37 °C in 5% CO_2_. The optical density of each well was measured at a wavelength of 450 nm using an EMax Endpoint ELISA Microplate Reader (Molecular Devices, Sunnyvale, CA, USA).

### 2.5. ROS Measurement

The ROS measurement was conducted with cellular a ROS Assay kit according to the manufacturer’s instructions. In brief, the HCEMC (2.5 × 10^3^ cells/well) were seeded onto a 96 well plate. The next day, the medium was removed and 1x buffer was added. After removal of 1x buffer, the cells were stained with DCFDA solution for 45 min at 37 °C in the dark. Just after incubation of the cells, DCFDA solution was removed and 1x PBS was added. The optical density of each samples was measured at Ex/Em = 485/535 nm at the end point using a MMR SPARK microplate reader (Tecan, Männedorf, Switzerland)

### 2.6. NO Quantification

The amount of NO in the CMEC-conditioned medium was quantified using the Griess reagent system, and the absorbance was measured at 540 nm using an EMax Endpoint ELISA Microplate Reader (Molecular Devices). The amount of nitrite in the culture media was calculated using sodium nitrite as a reference standard.

### 2.7. Preparation of Cell Extracts and Western Blot Analysis

The cells were rapidly washed twice with cold 1 × PBS and lysed with 1 × lysis buffer with 2 mM PMSF. The cells were harvested by scraping, and the supernatants were collected by centrifugation (rotor radius: 70 mm) at 13,500× *g* for 20 min. Protein concentrations of lysates were determined using a bicinchoninic acid (BCA) protein assay kit (Thermo Fisher Scientific, Rockford, IL, USA). Lysates were separated by SDS-PAGE and transferred to a nitrocellulose membrane. Blocking of the membrane was performed with 5% skim milk for 1 h. After blocking, the membranes were incubated with primary antibodies to detect Akt, phospho-Akt, eNOS, phospho-eNOS^ser1177^, GSK-3β, phospho-GSK-3β, and GAPDH at 4 °C overnight. The membranes were incubated with anti-immunoglobulin G horseradish peroxidase-conjugated secondary antibodies. The membranes were developed using EZ-Western Lumi Pico (Dogen, Seoul, Korea). Expression levels were quantified using Image J software.

### 2.8. Cytokine Array

The amount of cytokine in the CMEC-conditioned medium was measured by using a human angiogenesis array kit (R&D Systems) according to the manufacturer’s instructions. In brief, the array membrane was blocked with blocking buffer for 1 h at RT. After blocking, the membrane was incubated with detection antibody cocktail overnight at 4 °C. The next day, the membrane was incubated with streptavidin-HRP solution and visualized with chemi-reagent solution. Expression of each cytokine level was quantified with the Image J program. The list of cytokines tested is shown in Figure S1.

### 2.9. Tube Formation Assay

Subsequently, 10 uL of Matrigel were evenly distributed to each well of a μ-slide. CMECs (6.0 × 10^3^) were suspended with culture medium and seeded on Matrigel. The μ-slide was incubated at 37 °C with 5% CO_2_ for 5 h. The images were obtained using a microscope (Nikon Eclipse). For quantification of the ability to make a tubular structure, the net/ring structure was counted as a mesh. Average edge length of the mesh was regarded as a segment. The points that make up a segment were counted as junctions. The number of junctions, meshes, segments, and total length were measured using Image J software.

### 2.10. Statistical Analysis

All data are presented as the mean ± standard deviation (SD) of more than three independent experiments. P values of less than 0.05 were considered statistically significant. Statistical analysis of the data was carried out using an unpaired, two-tailed Student’s *t*-test.

## 3. Results

### 3.1. HG Treatment Induced Cardiac Microendothelial Dysfunction

**Figure 1:**
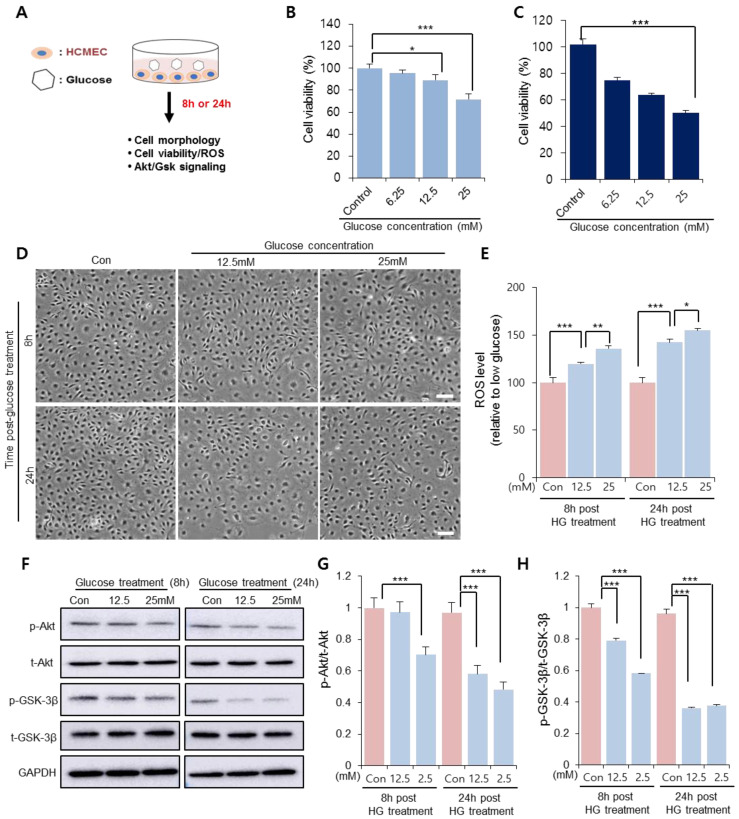
HG conditions impair viability of cardiac microendothelial cells. (**A**) Experimental scheme for glucose treatment. CMECs were exposed to HG conditions. (**B**,**C**) Cell viability and cellular morphology were observed at 8 and 24 h after treatment. Cell viability of CMECs was evaluated via WST-1 assays at (**B**) 8 and (**C**) 24 h. (**D**) Cellular shape was observed. Scale bar: 100 µm. (**E**) Reactive oxygen species production was quantified from supernatants of CMECs treated with HG. (**F**–**H**) p-Akt and p-GSK-3β were detected and represented as values relative to total Akt and GSK-3β, respectively. Expression levels were represented relative to that of the control. Values of *p* < 0.05 were considered significant (* *p* < 0.05, ** *p* < 0.01, *** *p* < 0.001). The data are expressed as the mean ± standard deviation of three independent experiments. CMECs, cardiac microvascular endothelial cells; HG, high glucose; Akt, protein kinase B; p-Akt, phosphorylated Akt; t-Akt, total Akt; GSK-3β, glucose synthase kinase 3 beta; GAPDH, glyceraldehyde-3-phosphate dehydrogenase; Con, control growth media.

To determine the glucose concentration that reduced CMEC viability, CMECs were treated with different concentrations of glucose in vitro prior to SP treatment (Figure 1A). After 8 and 24 h of treatment, the cellular morphology and viability were assessed via WST-1 assays. At 8 h after glucose treatment, 12.5 mM glucose slightly reduced cell viability and 25 mM glucose considerably reduced cell viability, compared to the control (Figure 1B; Control: 100 ± 3.6%, 6.25 mM: 95.4 ± 3%, 12.5 mM: 89.05 ± 5.24%, 25 mM: 71.62 ± 4.88%; 0 vs. 12.5 mM: *p* < 0.05, 0 vs. 25 mM: *p* < 0.005). This phenomenon was also observed at 24 h, when the cell viability was inversely proportional to the glucose concentration (Figure 1C). Therefore, the glucose concentration >12.5 mM was considered to affect CMEC function within 8 h, and the effect of 12.5 and 25 mM glucose on CMEC activity was subsequently evaluated.

In the presence of 12.5 mM and 25 mM glucose, the CMECs exhibited considerable alterations in cellular morphology, with enlarged/spread cell size and a few spindle cell shapes, which worsened at 24 h (Figure 1D). Free radical production in CMECs was quantified as the main effector of oxidative stress. ROS production increased as the glucose concentration increased at 8 h after treatment, and this effect was much stronger at 24 h (Figure 1E). It was confirmed that HG treatment decreased cellular activity under oxidative stress.

Akt/glycogen synthase kinase 3 beta (Gsk 3β) signaling is involved in the modulation of cell viability and apoptosis. HG treatment induces excessive ROS production, and oxidative stress can suppress Akt signaling. Therefore, we analyzed whether the cell viability reduced via the HG treatment of CMECs was accompanied by the inactivation of Akt/Gsk. Treatment with both concentrations of glucose showed a reduction in phosphorylated Akt and Gsk-3β levels in CMECs; however, 25 mM glucose considerably reduced the levels of active Akt/Gsk 3β at 8 h post-treatment (Figure 1F,G). This result suggests that HG conditions may affect the cell viability and morphology of CMECs, and accompanied by an increased ROS production and disrupted Akt phosphorylation. Based on the data shown in Figure 1, 25 mM glucose was found to be detrimental to CMECs, and this concentration was used in further experiments to determine the effect of SP on injured endothelial cells.

### 3.2. SP Treatment Blocked the Injury of CMECs Induced via HG Treatment and Reversed the Imbalance in Free Radical Production

**Figure 2:**
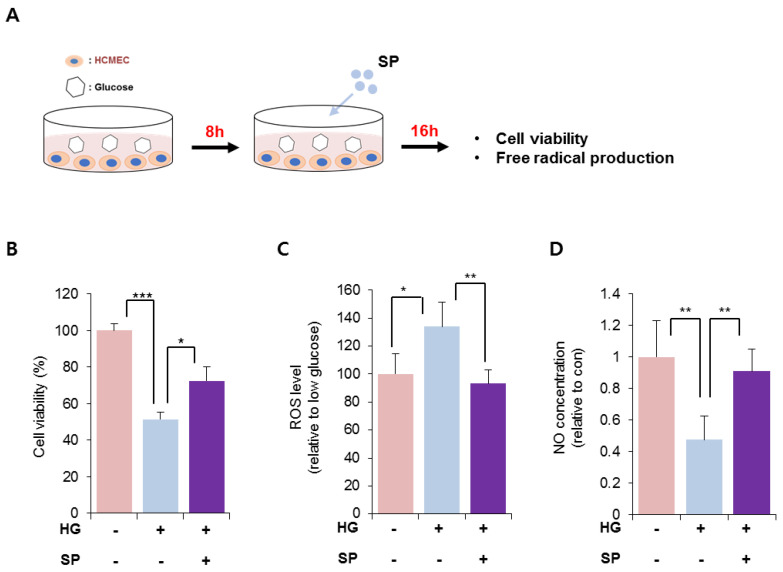
SP treatment restores the cellular activity of cardiac microendothelial cells with inhibition of the imbalance of free radicals. (**A**) Experimental scheme for glucose and SP treatment. (**B**) Cell viability of CMECs was evaluated via WST-1 assay. (**C**,**D**) Free radical generation was evaluated. (**C**) ROS and (**D**) NO production was represented relative to that in normal condition. Values of *p* < 0.05 were considered significant (* *p* < 0.05, ** *p* < 0.01, *** *p* < 0.001). The data are expressed as the mean ± standard deviation of three independent experiments. SP, substance-P; HG, high glucose; CMECs, cardiac microvascular endothelial cells; ROS, reactive oxygen species; NO, nitric oxide; con, control.

HG treatment reduced the viability of CMECs after 8 h. To explore the effect of SP on injured CMECs, CMECs were exposed to 25 mM glucose for 8 h and then treated with SP. At 24 h after treatment with 25 mM glucose, the cellular activity of CMECs was evaluated by analyzing the viability and free radical production (Figure 2A). Treatment with 25 mM glucose lowered the cell viability, which was reversed via SP treatment (Figure 2B; 25 mM glucose: 51.4 ± 4.03%, 25 mM glucose + SP: 72.38 ± 7.8%; *p* < 0.05). The effect of SP on cell viability was confirmed by TUNEL staining (Figure S2). Additionally, HG treatment increased ROS and decreased RNS production in CMECs, leading to an imbalance of free radicals. However, SP treatment reversed this change to maintain a balance of radicals, similar to that in control cells (Figure 2C,D). Moreover, SP treatment elevated the level of phosphorylated endothelial nitric oxide synthase (eNOS) that was reliably decreased by HG (Figure S3). This might be related to the recovery of NO in CMEC by SP treatment.

### 3.3. SP Treatment Could Restore Active Akt/Gsk 3β Signaling in CMECs under HG Stress Conditions

**Figure 3:**
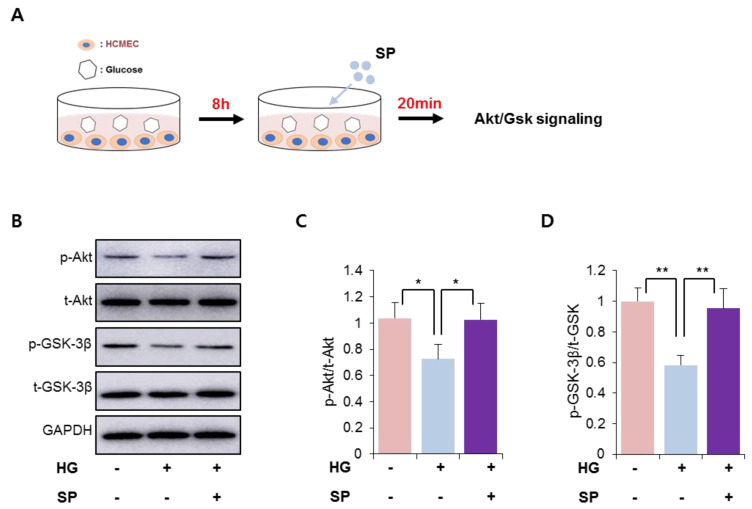
SP treatment activates PI3/Akt signaling in cardiac microendothelial cells exposed to HG. (**A**) Experimental scheme for glucose and SP treatment. (**B**–**D**) p-Akt and p-GSK-3β were detected via western blotting and the expression level was quantified. Values of *p* < 0.05 were considered significant (* *p* < 0.05, ** *p* < 0.01). The data are expressed as the mean ± standard deviation of three independent experiments. HG, high glucose; SP, substance-P; Akt, protein kinase B; p-Akt, phosphorylated Akt; t-Akt, total Akt; GSK-3β, glucose synthase kinase 3 beta; GAPDH, glyceraldehyde-3-phosphate dehydrogenase.

We found that HG treatment decreased the levels of phosphorylated Akt/Gsk 3β in CMECs (Figure 1). SP is known to activate the phosphoinositide 3-kinase (PI3K)/Akt signaling pathway, resulting in anti-apoptosis in diverse cell types [25,26]. We found that SP treatment could ameliorate the dysfunction in CMECs mediated via HG conditions by preserving cell viability and balancing free radical production (Figure 2). Thus, the effect of SP treatment was expected to be involved in the activation of Akt/Gsk 3β signaling in CMECs injured due to HG stress. To verify this, SP was added to CMECs that were incubated with HG for 8 h, and then the early activation of Akt/Gsk 3β was examined (Figure 3A). Under HG conditions, a reduction of phosphorylated Akt and Gsk 3β levels was consistently observed in CMECs, and SP treatment increased the levels of phosphorylated Akt and Gsk 3β that were reduced via HG treatment, to a level similar to that of control cells (Figure 3B–D). This indicates that SP may be capable of activating the PI3K/Akt signaling repressed by HG-mediated oxidative stress in CMECs, which may contribute to the recovery of cell viability via SP treatment.

### 3.4. HG Treatment Altered the Paracrine Potential of CMECs, Which Was Reversed via SP Treatment

Under normal physiological conditions, vascular endothelial cells play a role in angiogenesis via migration and proliferation. This is achieved by the release of several vasoactive molecules. However, HG-mediated production of cellular stress may induce the expression of various factors that promote inflammation or interrupt blood flow, which is considered to lead to the development of critical vascular diseases [34].

**Figure 4:**
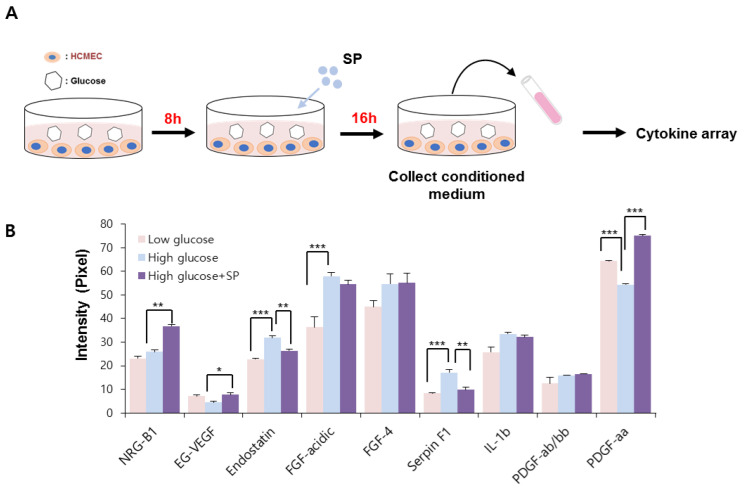
SP treatment prevents alteration of cytokine profile in cardiac microendothelial cells after high glucose treatment. (**A**) Experimental scheme for glucose and SP treatment. (**B**) Angiogenesis-related cytokine profile was analyzed via cytokine arrays. The relative expression was quantified using image J. Values of *p* < 0.05 were considered significant (* *p* < 0.05, ** *p* < 0.01, *** *p* < 0.001). The data are expressed as the mean ± standard deviation of three independent experiments. SP, substance-P; NRG-B1, neuregulin beta 1; EG-VEGF, endocrine gland-derived vascular endothelial growth factor; FGF, fibroblast growth factor; IL-1b, interleukin 1 beta; PDGF, platelet-derived growth factor.

In this study, the effect of SP on the paracrine pattern of CMECs was analyzed using a cytokine array. The conditioned medium was collected as shown in Figure 4. The relative expression of angiogenesis-related proteins was assessed, and those factors that were construed as being affected by HG or SP were quantified by image J. We found that the HG condition considerably affected the secretion of several angiogenesis-modulating factors, compared to in low-glucose conditions.

HG treatment increased the production of endostatin, fibroblast growth factor (FGF)-acidic, FGF-4, serphin F1, and interleukin (IL)-1 beta, and decreased the production of endocrine gland-derived vascular endothelial growth factor (EG-VEGF) and platelet-derived growth factor (PDGF)-aa in CMECs. Regarding the angiogenic potential, endostatin is an endogenous inhibitor of angiogenesis and interferes with the pro-angiogenic action of growth factors such as VEGF. Serphin F1 is a multifunctional protein with neurotrophic and anti-angiogenic properties. EG-VEGF, IL-1 beta, and PDGF-aa are well-known positive regulators of angiogenesis [35,36,37]. Therefore, HG conditions mediated the biochemical alteration of CMECs by affecting both angiogenic and anti-angiogenic factors. This effect might contribute to the development of a pathological environment in diabetes. However, SP treatment reversed this change in CMECs by increasing the expression of angiogenic factors and reducing anti-angiogenic factors via enrichment of EG-VEGF and PDGF-aa, and inhibition of the production of endostantin and serphin-F1. Neuregulin-1 is a positive regulator of angiogenesis and is closely related to myocardial angiogenesis [38], and its level is not correlated with HG-induced oxidative stress; however, its secretion is increased via SP treatment. Therefore, SP-mediated biochemical changes are expected to enhance the angiogenic potential of CMECs by providing an environment favorable for angiogenesis (Figure 4B). These data suggest that HG conditions may induce dysfunction in CMECs with disrupted paracrine potential; however, SP treatment induces CMEC skewing to enhance angiogenesis.

### 3.5. SP Treatment Enhanced the Ability to Develop Vessel-Like Structures in CMECs Injured by HG-Induced Oxidative Stress

**Figure 5:**
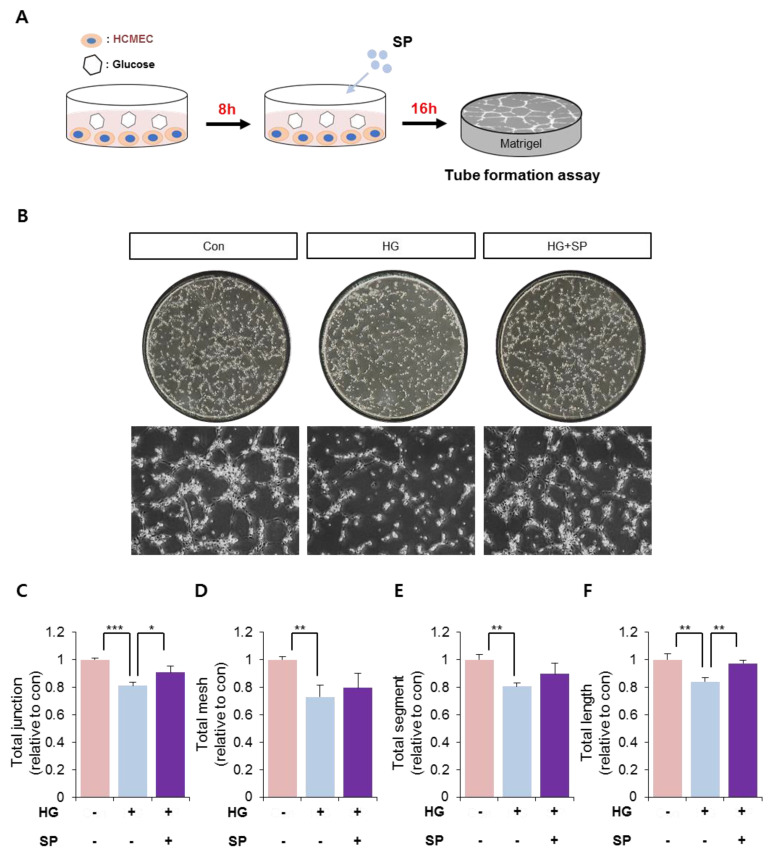
Hyperglycemia impairs the vascular structure-forming ability, which is restored via SP treatment. (**A**) Experimental scheme for glucose and SP treatment. (**B**) A representative image of CMECs cultivated on Matrigel. CMECs were subjected to Matrigel assays and the tube-forming ability was evaluated for 5 h. (**C**–**F**) Tube structures formed by CMECs were represented by total junction, mesh, segment, and length. Values of *p* < 0.05 were considered significant (* *p* < 0.05, ** *p* < 0.01, *** *p* < 0.001). The data are expressed as the mean ± standard deviation of three independent experiments. HG, high glucose; SP, substance-P; Con, control; CMECs, cardiac microvascular endothelial cells.

The main function of vascular endothelial cells is to develop the vasculature and maintain a stable state for circulation. SP treatment could increase cell viability and preserve the angiogenic potential in CMECs under HG-induced oxidative stress. Therefore, the effect of SP treatment on tube formation in CMECs was examined next. CMECs were treated with HG and SP, as shown in Figure 5A, and then subjected to a tube formation assay in vitro.

The control group showed the formation of tube-like structures, whereas HG-exposed CMECs did not form tube-like structures and showed a poor stretching. However, SP treatment induced tube formation in CMECs (Figure 5B). The effect of HG and SP on tube formation was quantified based on criteria including total length, junctions, and segments (Figure 5C–F). We observed that SP-treated CMECs could develop a compact vasculature with long stretching and tight junctions; therefore, SP treatment may inhibit the loss of tube formation induced by HG conditions. Tube formation is mainly mediated by autocrine soluble factors; thus, the SP-enhanced tube formation in CMECs may be due to a change in the secretory molecule pattern resulting from SP treatment.

## 4. Discussion

The development of hyperglycemia is observed in cases of diabetes (both type 1 and type 2). Hyperglycemia promotes ROS accumulation, which induces cellular damage. Hyperglycemia-induced oxidative stress induces endothelial dysfunction, which plays a central role in the pathogenesis of micro- and macro-vascular diseases [18,19]. It may also upregulate pro-inflammatory and pro-coagulant factors, induce apoptosis, and impair the release of NO [39]. These events are associated with the development of diabetic complications induced by hyperglycemia [40].

In this study, HG conditions were found to reduce the cellular activity of CMECs with a phenotypic alteration. During this process, HG conditions could increase ROS production and decrease NO secretion in CMECs, leading to an imbalance in free radical formation. To maintain cell viability and active metabolism, survival-related signaling is activated to modulate cell proliferation/apoptosis-related gene expression. However, CMECs exposed to HG show low levels of active Akt/GSK-3β, which are repressed to an indiscernible level over time. This may be associated with the poor viability of CMECs after HG treatment.

SP has been previously reported to protect cell apoptosis against inflammation or oxidative stress in the retina and adipose stem cells [25,26,27], and the systemic administration of SP has been shown to relieve diabetic complications in preclinical studies [28]. However, its mechanism of action on the endothelium has not yet been elucidated. In this study, CMECs damaged by HG conditions were preferentially treated with SP, and the effects of SP were evaluated in terms of cell viability and endothelial function. HG treatment for a period of 8 h clearly reduced CMEC viability, which was reversed via SP treatment. This effect was accompanied by decreased ROS levels and increased NO production/eNOS activation in CMECs. Moreover, SP could activate PI3/Akt and GSK-3β signaling in HG-treated CMECs. Therefore, SP treatment could create a restorative environment for CMECs injured by hyperglycemia.

Angiogenesis and vasculogenesis are mainly regulated by soluble factors. Therefore, preservation of the paracrine function of endothelial cells is important for vascular function and for structural support of the vessel. This study evaluated the cytokine profile of CMECs under normal glucose and hyperglycemic conditions for the first time. HG conditions regulated the production of angiogenesis-related factors and affected the formation of tube-like structures in CMECs. These events suggest the development of a distinct dysfunction of endothelial cells, inducing the development of diabetic complications. However, SP treatment mitigated the HG-induced alterations. In particular, SP treatment mediated the development of a cellular state with angiogenic potential by inducing positive regulators of vessel formation, which contributed to well-organized vasculature formation in CMECs in vitro. The blockage of endothelial dysfunction via SP treatment is expected to play a role in the remission of diabetic complications.

In summary, we found that hyperglycemia induced CMEC dysfunction by reducing cell viability and inducing morphological alterations, ROS production, altered cytokine profiles, and poor vascular structure-forming capacity. However, when the injured CMECs were treated with SP, the deterioration of CMECs by HG conditions was reversed and the cellular function and viability of the CMECs were restored. This study is fundamental for determining the in vivo efficacy of SP in diabetes cases. The therapeutic potential of SP for treating CMECs remains to be explored. The molecular mechanism of SP-mediated secretome production in the endothelium will be explored in further studies.

## Data Availability

The datasets used and/or analyzed during the present study are available from the corresponding author on reasonable request.

## Supplementary Materials

The following are available online at https://www.mdpi.com/article/10.3390/antiox10071084/s1, Figure S1: The list of cytokine tested in cytokine array; Figure S2: The effect of SP on cell apoptosis under HG; Figure S3: The analysis for eNOS expression in CMEC under HG.
