# Supplementary material for: Substance-P Inhibits Cardiac Microvascular Endothelial Dysfunction Caused by High Glucose-Induced Oxidative Stress
Source: Antioxidants (Basel). 2021 Jul 5;10(7):1084. doi: 10.3390/antiox10071084 (PMC8301094; doi:10.3390/antiox10071084)
Supplement: Supplementary file 1 [file antioxidants-10-01084-s001.zip › antioxidants-1278194-supplementary.pdf]

Activin A	FGF-7/KGF	PD-ECGF	ADAMTS-1	GDNF	PDGF-AA
Angiogenin	GM-CSF	PDGF-AB/PDGF-BB	Angiopoietin-1	HB-EGF	Persephin
Angiopoietin-2	HGF	CXCL/PF4	Angiostatin/Plasminogen	IGFBP-1	PIGF
Amphiregulin	IGFBP-2	Prolactin	Artemin	IGFBP-3	Serpin B5/Maspin
Tissue Factor/Factor III	IL-1 beta	Serpin E1/PAI-1	CXCL16	CXCL8/IL-8	Serpin F1/PEDF
DPPIV/CD26	LAP (TGF-beta 1)	TIMP-1	EGF	Leptin	TIMP-4
EG-VEGF	CCL2/MCP-1	Thrombospondin-1	Endoglin/CD105	CCL3/MIP-1 alpha	Thrombospondin-2
Endostatin/Collagen XVIII	MMP-8	uPA	Endothelin-1	MMP-9	Vasohibin
FGF acidic	NRG1-beta 1	VEGF	FGF basic	Pentraxin 3	VEGF-C
FGF-4					

Figure S1. The list of cytokine tested in cytokine array.

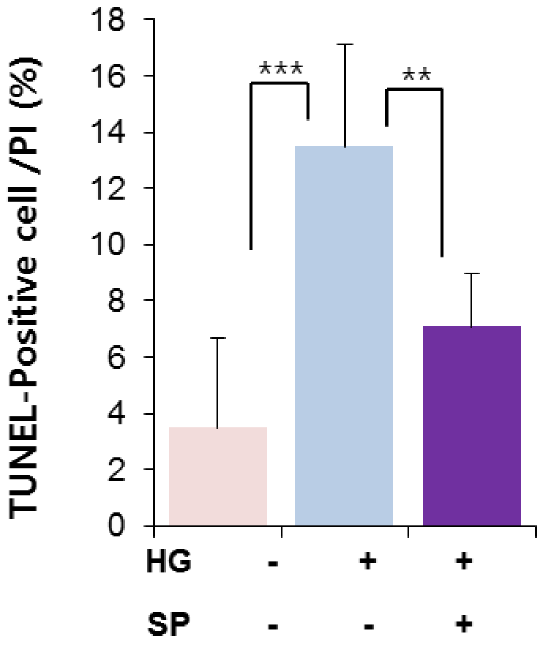

Figure S2. The effect of SP on cell apoptosis under HG. CMEC was exposed to HG for 8h, followed by SP treatment. At 24h post HG treatment, cell was fixed and stained for apototic cell using in situ cell death detection kit. Total cell was stained with Propidium iodide (PI) and TUNEL (+) cells were expressed as a percentage of PI-stained cells.

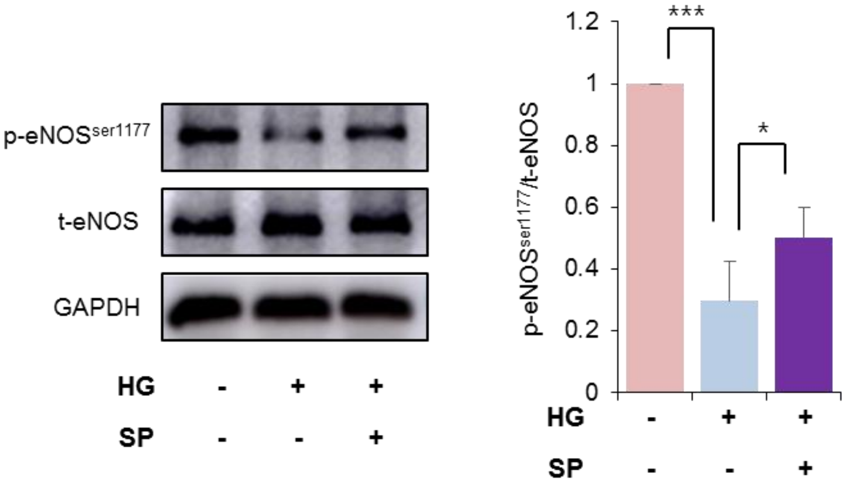

Figure S3. The analysis for eNOS expression in CMEC under HG.CMEC was exposed to HG for 8h, followed by SP treatment. At 24h post HG treatment, cell lysate was prepared and phosphorylated eNOS and total eNOS were analyzed by western blot.
